# Supplementary material for: Availability, affordability and stock-outs of commodities for the treatment of snakebite in Kenya
Source: PLoS Negl Trop Dis. 2021 Aug 16;15(8):e0009702. doi: 10.1371/journal.pntd.0009702 (PMC8389522; doi:10.1371/journal.pntd.0009702)
Supplement: S1 Table — (DOCX) [file pntd.0009702.s001.docx]

**Supporting Information Table 1. Surveyed snakebite commodities.**

| **Commodity** | **Formulation** | **Level of care available** | **Use** |
| --- | --- | --- | --- |
| Antivenom | 10ml | 2 | Specific antibody treatment for envenomings. |
| Tetanus toxoid vaccine | 10ml | 2 | Prevention of tetanus. |
| Benzylpenicillin | 600mg | 2 | Prevention of bacterial infections. |
| Metronidazole | 200mg, 400mg | 2 | Prevention of bacterial infections. |
| Metronidazole | 200mg/5ml | 4 | Prevention of bacterial infections. |
| Gentamicin | 10mg/2ml, 20mg/2ml | 2 | Prevention of bacterial infections. |
| Gentamicin | 40mg/2ml, 80mg/2ml | 3 | Prevention of bacterial infections. |
| Amoxicillin | 250mg, 500mg | 2 | Prevention of bacterial infections. |
| Amoxicillin + clavulanic acid | 250mg + 62.5mg,  875mg + 125mg | 2 | Prevention of bacterial infections. |
| Adrenaline | 1mg/ml | 2 | Treatment of anaphylaxis. |
| Hydrocortisone | 100mg | 2 | Treatment of serum sickness. |
| Chlorpheniramine | 10mg/1ml, 2mg/5ml | 2 | Treatment of serum sickness. |
| Prednisolone | 5mg | 4 | Treatment of serum sickness. |
| Neostigmine | 2.5mg/ml | 4 | Reversion of neuromuscular blockage |
| Atropine | 1mg/ml | 4 | Reversion of neuromuscular blockage |
| Paracetamol | 500mg | 1 | Management of mild pain. |
| Dihydrocodeine phosphate | 30mg | 3 | Management of moderate to severe pain. |
| Morphine | 10mg/ml | 2 | Management of severe pain. |
| Lidocaine | 30ml | 2 | Topical anaesthetic. |
| Saline (sodium chloride) | 500ml | 2 | Treatment of anaphylaxis/circulatory failure. |
| Fresh frozen plasma | NA | 4 | Blood transfusion. |
| Blood platelets | NA | 4 | Blood transfusion. |
| Red blood cells | NA | 4 | Blood transfusion. |
| Whole blood | NA | 4 | Blood transfusion. |
| Bandage | NA | 1 | Bind up a wound or part of the body. |
| Splint | NA | 3 | Pressure immobilization. |
| Sticking plaster | NA | 3 | Pressure immobilization. |
| Oxygen cylinder | NA | 2 | Provision of oxygen to patients. |
| Laryngoscope | NA | 2^a^ | Examination of the throat, insertion of tube into throat. |
| Cuffed endotracheal tube | NA | 3 | Maintains an open airway to provide oxygen. |
| Nasal prong | NA | 2 | Deliver supplemental oxygen through the nose. |
| Ambu bag | NA | 2 | Manual ventilation. |
| Oral airway | NA | 3 | Maintains an open airway. |
| Ventilator | NA | 3^a^ | Assist the function of the lungs of a patient. |
| Intravenous cannula | NA | 2 | Administration of fluids and medicines. |
| Catheter | NA | 2 | Drainage of bladder. |
| Syringe + needle | NA | 2 | Administration of fluids and medicines. |
| IV administration set | NA | 2 | Administration of fluids and medicines. |
| Urine dipstick | NA | 2 | Envenoming test. |
| Creatinine clearance blood test | NA | 2^a^ | Envenoming test. |
| Blood urea nitrogen testing | NA | 2^a^ | Envenoming test. |
| 20-minute whole blood clotting test | NA | 2^a^ | Envenoming test. |
| Point-of-Care INR device | NA | 2^a^ | Envenoming test. |
| IV: Intravenous; INR: International normalised ratio; NA: Not applicable.  Level 1: Community health services; level 2: Dispensary/clinic; level 3: Health centre; level 4: Primary hospital; level 5: Secondary hospital; level 6: Tertiary hospital.  ^a^Commodity not included on essential medicines or medical supplies list. | | | |
