# Supplementary material for: Availability, affordability and stock-outs of commodities for the treatment of snakebite in Kenya
Source: PLoS Negl Trop Dis. 2021 Aug 16;15(8):e0009702. doi: 10.1371/journal.pntd.0009702 (PMC8389522; doi:10.1371/journal.pntd.0009702)
Supplement: S2 Table — (DOCX) [file pntd.0009702.s002.docx]

**Supporting Information Table 2. Antivenom brands stocked at health facilities, per sector.**

| **Brand** | **Number of Facilities** | | |
| --- | --- | --- | --- |
|  | **Public** | **Private** | **Total^a^** |
|  | N (%) | N (%) | N (%) |
| **Snake Venom Antiserum (African IHS)**  VINS Bioproducts Ltd | 24 (66.7) | 6 (100.0) | 30 (70.2%) |
| **Inoserp PAN-AFRICAN**  INOSAN Biopharma | 13 (33.3) | 0 (0.0) | 13 (29.8%) |
| **SAIMR Polyvalent Snake Antivenom**  South African Vaccine Producers (SAVP) (PTY) Ltd | 1 (2.6) | 0 (0.0) | 1 (2.1%) |
| **Brand information missing** | 1 (2.6) | 0 (0.0) | 1 (2.1%) |
| ^a^Availability includes the private not-for-profit sector. | | | |
