# Supplementary material for: Availability, affordability and stock-outs of commodities for the treatment of snakebite in Kenya
Source: PLoS Negl Trop Dis. 2021 Aug 16;15(8):e0009702. doi: 10.1371/journal.pntd.0009702 (PMC8389522; doi:10.1371/journal.pntd.0009702)
Supplement: S3 Table — (DOCX) [file pntd.0009702.s003.docx]

**Supporting Information Table 3. Accessibility of snakebite commodities, per sector.**

|  | Public | |  | Private | |
| --- | --- | --- | --- | --- | --- |
|  | Availability | Affordability |  | Availability | Affordability |
| Antivenom | 44.7% | 0.0 |  | 19.4% | 14.4 |
| Tetanus vaccine | 68.2% | 0.0 |  | 83.3% | 0.2 |
| Benzylpenicillin | 63.5% | 0.0 |  | 69.4% | 4.4 |
| Metronidazole (200 or 400mg) | 83.5% | 0.0 |  | 75.0% | 0.2 |
| Metronidazole (200mg/5ml) | 20.0% | 0.0 |  | 30.6% | 0.1 |
| Gentamicin (10mg/2ml or 20mg/2ml) | 37.6% | 0.0 |  | 8.3% | 2.2 |
| Gentamicin (40mg/2ml or 80mg/2ml) | 55.3% | 0.0 |  | 61.1% | 0.6 |
| Amoxicillin (250mg) | 87.1% | 0.0 |  | 77.8% | 0.2 |
| Amoxicillin (500mg) | 34.1% | 0.0 |  | 69.4% | 0.3 |
| Amoxicillin + clavulanic acid | 51.4% | 0.0 |  | 37.9% | 1.0 |
| Paracetamol | 88.2% | 0.0 |  | 97.2% | 0.0 |
| Dehydrocodeine phosphate | 4.2% | 0.0 |  | 3.4% | N/A |
| Morphine | 15.8% | 0.2 |  | 18.2% | 1.1 |
| Adrenaline | 43.5% | 0.0 |  | 61.1% | 0.2 |
| Hydrocortisone | 80.0% | 0.0 |  | 77.8% | 1.3 |
| Chlorpheniramine (10mg/1ml) | 9.7% | 0.1 |  | 37.9% | 0.7 |
| Chlorpheniramine (2mg/5ml) | 8.3% | 0.0 |  | 24.1% | 0.3 |
| Prednisolone (5mg) | 38.8% | 0.0 |  | 86.1% | 0.1 |
| Saline | 81.2% | 0.0 |  | 63.9% | 0.6 |
| Lidocaine | 75.3% | 0.0 |  | 75.0% | 0.0 |
| Neostigmine | 21.1% | 0.1 |  | 18.2% | 0.5 |
| Atropine | 52.8% | 0.0 |  | 58.6% | 0.2 |
| Accessibility:  <80% availability, >1 day’s wage <80% availability, <1 day’s wage  ≥80% availability, >1 day’s wage ≥80% availability, <1 day’s wage | | | | | |
