# Supplementary material for: Availability, affordability and stock-outs of commodities for the treatment of snakebite in Kenya
Source: PLoS Negl Trop Dis. 2021 Aug 16;15(8):e0009702. doi: 10.1371/journal.pntd.0009702 (PMC8389522; doi:10.1371/journal.pntd.0009702)
Supplement: Attachment — Submitted filename: Response to reviewers.docx [file pntd.0009702.s004.docx]

***Methods***

**Reviewer #1**

1. While there have been many anecdotal reports of antivenom access challenges, the manuscript is one of the very rare examples to measure objectively access to essential products for management of snakebite envenoming, and particularly access to antivenom. The survey method is very clear. healthcare facilities in 6 health counties were surveyed. The sample is large enough to measure significant differences. The list of surveyed items was defined by snakebite experts and was based on national and international guidelines. It reflects (to one notable exception described below) the list of commodities that are needed in a country like Kenya for effective management of snakebite envenoming. The method to evaluate product affordability is robust and has been used in the past for other medicines. I would simply recommend to better describe what "availability" and "stock out" exactly mean in this study (see below).

We thank the reviewer for the feedback. We have now more clearly described what is meant with ‘availability’ and ‘stock-out’: “Availability was defined as the presence of a survey medicine in pre-specified dose and formulation at the time of the data collection in the health facility.” (methods section, p.9, line 163-164); “A commodity was considered stocked out if the facility usually stocked the commodity, but the stock-taking database indicated it had been out of stock at times in the past year.” (methods section, p.10, line 189-191).

**Reviewer #2**

1. The objective of the study was clearly stated. However, the authors need to state how they arrive at 6 survey regions. The authors adapted non-probability sampling method this could have been improved by using multi-stage sampling method initially and later probability sampling method. 24 health facilities were randomly selected, from a pool of what number of health facilities. The denominator need to be stated. Why was community health services (level 1) excluded from the survey, they provide first aid services in rural communities and this can improve survival of snakebite victims.

We thank the reviewer for raising these points. The methodology used is a gold-standard that has been validated in many countries over the years. Per this methodology, six survey regions are selected for inclusion, and in each region 24 facilities are selected. Level I services were excluded from the study because most of the commodities surveyed are not stocked at this level. We have tried to clarify the sampling strategy used: “This study adopted a quantitative cross-sectional survey design with a retrospective component, using an adapted version of the standardised, gold-standard WHO/Health Action International (HAI) methodology measuring the availability, stock-outs and affordability of commodities (18). Per this methodology, in six survey regions, 24 health facilities were randomly selected from the public, private, and private not-for-profit (PNFP) sectors, in both urban and rural locations, to function as a representative sample. A rural location was defined as an area with a population of less than 2,000 people (19). This sampling strategy has been validated in many countries (18,20). The six survey regions in this study were purposively sampled: four were highly snakebite endemic and HAI programme counties (Kajiado County, Kilifi County, Kwale County and Taita Taveta County), and two were less endemic (Kirinyaga County and Nyandarua County). Using the Kenya Master Health Facility List, in each county, the main public hospital was selected for inclusion, after which the other 23 licensed facilities were randomly selected under the prerequisite that they were within about an hour’s drive from the main public hospital (21). The master list used consisted of 52 facilities in Kajiado-, 26 in Kilifi-, 48 in Kirinyaga-, 40 in Kwale-, 33 in Nyandarua- and 26 in Taita Taveta County. The selected facilities were categorised according to sector and location. The levels of health facilities surveyed ranged from level 2: dispensaries and clinics to level 6: tertiary hospitals, thereby only excluding level 1: community health services, which are not expected to stock most of the commodities surveyed in this study.” (methods section, p.7-8, line 121-139). We have also added to the limitations that a non-probability sampling method was used (discussion section, p.38, line 513-514).

**Reviewer #3**

Introduction section:

1. Line 78 inter alia (typo)

The line now reads ‘inter alia’ (Introduction section, p. 5, line 78).

2. Line 79 change airway clearance to airway support

We thank the reviewer for the suggestion. ‘Airway clearance’ has now been changed to ‘airway support’ (introduction section, p. 5, line 79).

3. Line 80 term resuscitation is not specific. Is this cardiopulmonary resuscitation or fluid resuscitation? Suggest remove it as CPR is (unfortunately) rarely available in in settings without access to ITU.

We thank the reviewer for raising the point, and have adjusted accordingly; ‘resuscitation’ has now been removed (introduction section, p.5, line 81).

4. Line 114 suggest removing term 'first wave'. The epidemiology of COVID in Kenya has been quite different to Europe and the USA and I don't think it is possible to refer to distinct waves when transmission has been fairly constant in Kenya.

We agree with the reviewer that calling it the first wave is inappropriate. The sentence now reads: “This research provided a unique and timely opportunity to study stock-outs of snakebite commodities in Kenya during the first few months of the COVID-19 pandemic.” (introduction section, p.7, line 115-117).

Methods section:

5. I would suggest adding an aim(s) section to the methods

We thank the reviewer for the suggestion. Instead of adding the aims to the methods, we have clarified the aim of the study in the introduction, where the aims of a research are more commonly described: “The aim of this study was therefore to determine the availability, affordability, and stock-outs of antivenom and commodities used for supportive snakebite treatment in health facilities across Kenya to build the evidence needed to take targeted action to reduce the burden of snakebite.” (introduction section, p.6, line 106-109).

6. Line 122 was random selection of facilities stratified to ensure adequate numbers of each type of facility? And what data source was used to create a master list of facilities to randomize from? Would this list have captured all facilities?

Thank you to the reviewer for raising these points. We did not stratify the facilities into public, private, PNFP before random selection because of the unequal distribution of facilities. It is a trade-off between having more information about the private and PNFP sector, and the overall availabilities providing a more representative image of the situation, which now is the case. Per the HAI/WHO methodology, at least 5 facilities per sector should be surveyed per survey region. As we did not reach this number for the PNFP sector, we have not reported stratified availabilities/prices for this sector. We clarified the sampling strategy: “Per this methodology, in six survey regions, 24 health facilities were randomly selected from the public, private, and private not-for-profit (PNFP) sectors, in both urban and rural locations, to function as a representative sample. A rural location was defined as an area with a population of less than 2,000 people (19). This sampling strategy has been validated in many countries (18,20). The six survey regions in this study were purposively sampled: four were highly snakebite endemic and HAI programme counties (Kajiado County, Kilifi County, Kwale County and Taita Taveta County), and two were less endemic (Kirinyaga County and Nyandarua County). Using the Kenya Master Health Facility List, in each county, the main public hospital was selected for inclusion, after which the other 23 licensed facilities were randomly selected under the prerequisite that they were within about an hour’s drive from the main public hospital (21). The master list used consisted of 52 facilities in Kajiado-, 26 in Kilifi-, 48 in Kirinyaga-, 40 in Kwale-, 33 in Nyandarua- and 26 in Taita Taveta County. The selected facilities were categorised according to sector and location. The levels of health facilities surveyed ranged from level 2: dispensaries and clinics to level 6: tertiary hospitals, thereby only excluding level 1: community health services, which are not expected to stock most of the commodities surveyed in this study.” (methods section, p.7-8, line 124-139).

7. Line 147 - Need more detail about what the data collectors did. Did they visit every healthcare facility that was sampled? Did they check stocks and records themselves or interview staff?

We have clarified that the data collectors visited each facility, and only recorded information about the stocks and if they were physically seen (methods section, p.9, line 160-165).

8. Line 148 - Is the licensed healthcare worker someone employed by your study or by the facility?

The licensed healthcare worker was employed by the facility. We have clarified this in the paper (methods section, p.9, line 160-162).

9. Line 158 - remove term 'an analysis tool was used which included formulas to calculate...' Suggest change wording to 'Simple descriptive statistics were used to describe the availability of commodities, and results were stratified for public, private, rural and urban facilities.'

We thank the reviewer for the suggestion, and agree that the suggested change improves the readability. We have changed the sentence to: “Simple descriptive statistics were used to describe the availability and affordability of commodities, and results were categorised according to sector (public, private or PNFP), and location (urban or rural).” (methods section, p.9-10, line 174-178).

10. Line 166 - I don't understand what 'aggregate availability' means?

Aggregate availability is the combined availability of commodities which were surveyed in multiple formulations. Meaning if, for example, amoxicillin was only available in 500mg formulation, not in 250mg at a specific facility, the combined commodity of amoxicillin (any formulation) would still be classified as available. We have changed ‘aggregate’ to ‘combined’ to facilitate understandability (methods section, p.9, line 183-186).

11. Line 185 - I don't understand 'an 'X' was used to denote...

The sentence explains how data collectors could indicate that a commodity was available even if the price was unknown, as this was included in the survey as combined measure. We have decided to delete the sentence: “When prices were not provided by the health facilities, an ‘X’ was used to denote availability, without denoting any price” as it does not add significance to the section.

12. Line 188 specify that it is 452 KSH *per day*

Thank you to the reviewer for noting this. We have added ‘per day’ (methods section, p.11, line 210).

13. Line 190-191 is difficult to follow - suggest reword

We have tried to make the sentence easier to follow: “Second, since the LPGW measure knows some limitations with representativeness, as the wage of an LPGW is much higher than the income of a large proportion of the population, the impoverishment approach as developed and described by Van Doorslaer et al. (2006) was also used.” (methods section, p.11, line 212-216).

***Results***

**Reviewer #1**

1. Results are clearly presented. The discussion section does focus on the main take away messages. Both Figures 1 and 2 clearly present the main findings in terms of availability and affordability.

Thank you.

**Reviewer #2**

1. Statistical analysis not clearly presented, the tables should contain level of significance in terms of p-value/confidence interval were applicable.

We thank the reviewer for pointing this out. We have now adjusted the tables to include the p-values (results section, p.17-18, line 268), (results section, p.23-24, line 303).

**Reviewer #3**

2. No mention of the response rate. Did every facility that was randomly selected provide data? Should report this at beginning.

We have now added the number of facilities approached, as well as the participation rate (results section, p.13, line 242-245).

3. Line 230 - further detail about antivenom would be helpful. Can you provide a breakdown by facility type. I would not expect dispensaries to stock antivenom as they usually do not have the facilities to administer intravenous drugs, and certainly would not be a safe place to manage an anaphylactic reaction. I appreciate you state that they are expected to stock it, but it may be worth presenting stock availability in hospitals too. Could you also present the percentage of facilities that stock antivenom but do not stock adrenaline - as all facilities giving antivenom should be able to manage anaphylaxis.

Thank you to the reviewer for the suggestion. We have now expanded table 3 to also include the availability of both antivenom and adrenaline. We have also provided more information in-text about availability of antivenom in higher level facilities such as hospitals (results section, p.13, line 252-255), (results section, p.19, line 273).

4. Table 2 and 3 - it is quite challenging for the reader to scan the results and see where availability is particularly low. I wonder if you could shade the cells with a gradient to depict different levels of availability. For example, see heatmap Figure 1a of Ainsworth et al <https://doi.org/10.1371/>journal.pntd.0008579

We agree with the reviewer that adding a gradient to depict different levels of availability enables the reader to scan the results more easily. We have now added such a gradient to tables 2 and 3 (results section, p.17-19, line 268-273).

5. Table 4 - I was surprised to see stock-outs were as low as 20% for antivenom. And only 13 days of the year was antivenom not available. To me that suggests that those facilities that do have it have fairly consistent supply. Whereas 65% of facilities don't have it at all. This may be worth touching on in the discussion.

We thank the reviewer for the suggestion, and have now added a few sentences on this finding in the discussion: “Further, 20% of the facilities that did stock antivenom experienced stock-outs, which lasted on average 13.6 days per facility. This seems to suggest that while one-fifth of facilities experienced stock-outs of antivenom, the supply is more or less consistent, with stock-outs, although still troubling, not occurring for extended periods of time. Nevertheless, this study did not ask about the number of vials available at the facility. Since several vials are required per treatment per patient, the supply could be consistent for a period but not necessarily a supply to meet treatment demand. Focus should therefore be on making antivenom adequately available in facilities, especially where it is not yet stocked.” (discussion section, p.32-33, line 398-405).

6. Box 1 - Don't refer to black stone as first aid as this may suggest it is appropriate first aid. Perhaps 'traditional therapy.' Don't refer to use of hydrocortisone in first facility - this is inappropriate and shouldn't have been given. Highlight that after receiving antivenom the child developed an anaphylactic reaction that required treatment with adrenaline, hydrocortisone and chlorpheniramine. During the hospital stay the child developed a bite site soft tissue infection and sepsis that prompted administration of co-amoxiclav and gentamicin (note co-amoxiclav is given three times daily but gentamicin is only once daily). Paracetamol is usually given four times per day.

We agree with the reviewer that it is better to not label use of black stone as first aid, as it might confuse the reader about its use. We have now changed it to ‘traditional treatment’. We have also added a note that the hydrocortisone given in the first facility was inappropriate. No anaphylactic reaction had developed, but adrenaline was provided as premedication, so no hydrocortisone and chlorpheniramine were provided. The dosages for gentamicin and paracetamol have been adjusted. (results section, p.27-28, line 338)

7. Figure 1 - it is challenging to refer to the key for each dot on the plot. Could you use a cross table with affordability against availability. Consider heatmap as for Table 2/3.

We understand the reviewer’s point. However, we believe adding another large table to the paper will make it even longer, as there are already two large tables in the paper at the moment. Instead, we have tried to make the figures clearer by using leader lines for each point in the figure, and have added the table to the supplementary file (results section, p.29-30, line 351-358).

Discussion:

8. Line 405 - 'envenoming tests' is vague term. Suggest remove this sentence as the only test available in sub-Saharan Africa is 20MWBCT. Venom EIAs are only routinely used/available in Australia.

We have accepted the reviewer’s suggestion, and removed the part mentioning ‘envenoming tests’ (discussion section, p.35, line 450-453).

9. Line 420 'population' duplicated

Thank you for noting this duplication. We have omitted one ‘population’ (discussion section, p.36, line 467).

10. In the discussion I would highlight that VINS is not supported by pre-clinical data and Inoserp is probably more appropriate (referring to Harrison's paper as you have done). Although I would add the caveat that no antivenom used in Kenya is supported by data from a randomised controlled trial. Nevertheless it is worrying that facilities have still not shifted away from VINS.

We agree with the reviewer that it is important to highlight this problem, and have now done so: “This study further showed that Snake Venom Antiserum (African IHS) produced by VINS Bioproducts Ltd, and Inoserp PAN-AFRICAN produced by INOSAN Biopharma were the most commonly stocked antivenom products, which have been shown to be ineffective in preclinical tests for some of the most commonly found snakes in Kenya. Especially for the VINS antivenom, its use is not supported by any pre-clinical data (10). Important to further note is that no antivenom in use in Kenya is supported by data from a randomised controlled trial.” (discussion section, p.33, line 407-412).

**Conclusions**

**Reviewer #1**

1. The conclusions are supported by the data. This manuscript provides robust data at country level and a good baseline to measure progress in the coming years in Kenya.

Thank you to the reviewer for appreciating the research.

**Reviewer #2**

1. Conclusion supported by data presented

Thank you.

**Reviewer #3**

1. Line 488 - typo remove 'there'

‘There’ has now been removed. Thank you for noting the typo.

2. Line 492 - I dont feel notifiable is the correct term. Notifiable diseases are infectious and each case poses a risk to the wider public (such as a viral haemorrhagic fever). Agree with your assertion that data per facility should be routinely recorded to allow planning of stock. Perhaps 'routine national surveillance' would be a more appropriate term.

We thank the reviewer for the suggestion, and have accepted the change. The sentence now reads: To facilitate this, snakebite should be made a part of routine national surveillance, with mandatory recording of the number of snakebites admitted to health facilities at all levels. (discussion section, p.34, line 437-439).

**Editorial and Data Presentation Modifications**

**Reviewer #1**

A few points however require clarification in my opinion.

1. LIST OF SURVEYED HEALTH PRODUCTS. I don’t understand exactly why tetanus immunoglobulins are listed by your survey. Neither the WHOI guidelines on snakebite envenoming in Africa (your ref number 4); not the Kenyan guidelines (your ref number 20) seem to be recommending tetanus immunoglobulins for the management of snakebite. Tetanus toxoid (vaccine) is recommended but not the immunoglobulins. It may be more reasonable to exclude them from the list. For your consideration. [That being said, it was interesting to read that tetanus immunoglobulins wasn’t available in any of the surveyed facilities!]

Thank you to the reviewer for noting this. We have now removed anti-tetanus immunoglobulins from the analyses and paper.

2. DEFINITIONS OF "AVAILABILITY" AND "STOCK OUT". It is unclear to me what availability and unavailability exactly mean. Does it mean that the item was available /or stocked at the time of the survey? Maybe it would be useful to clarify.

We thank the reviewer for the feedback. We have now more clearly described what is meant with ‘availability’ and ‘stock-out’: “Availability was defined as the presence of a survey medicine in pre-specified dose and formulation at the time of the data collection in the health facility.” (methods section, p.9, line 163-164); “A commodity was considered stocked out if the facility usually stocked the commodity, but the stock-taking database indicated it had been out of stock at times in the past year.” (methods section, p.10, line 189-192).

3. Similarly, it may be useful to clarify early in the text that the occurrence of stock-outs over the last 6-12 months was monitored only among facilities where the item is available. For example, neostigmine is available in only 17.1% of facilities while stock outs have been reported in 40% of facilities. From my understanding, this means that 40% of the 17.1% facilities in which neostigimine is available reported a stock out in the last 12 months. Did I get it right? In any case, it may be useful to clarify.

Thank you to the reviewer for raising this point. Stock information was asked after for all commodities, not only for those that were in stock at the time of the survey, as it is possible the commodity was stocked out at that moment. This means that for neostigmine, 17.1% of all health facilities had the commodity available at the time of survey, and 40% of all health facilities which had stock information available for neostigmine experienced a stock out of neostigmine at least once in the past year. We have clarified this in the methods: “Stock-out information was asked for all commodities supposed to be available at that level of care, regardless of whether they were in or out of stock at the time of the survey.” (methods section, p.10, line 191-192).

4. DISPARITIES BETWEEN COUNTIES. Your analysis is based on the following dichotomies: private vs public and rural vs urban. I am however curious to know if there were disparities between counties. Kenya’s health system is very much decentralized, so I wouldn’t be surprised if some counties had better results than others. Maybe your study is not powered to measure that, since only 6 counties have been included. Yet, if you have meaningful data, I would like to encourage you to include them. If your data aren’t meaningful, then forget about my recommendation.

We thank the reviewer for the suggestion. We stratified the sample into the endemic and less endemic counties for antivenom, however, to split our data up per county separately is not recommended as only 21-24 health facilities were surveyed per county. Stratifying per county will then make the sample too small to be able to say something meaningful about the data. We therefore believe it is best to keep to the stratification used now.

5. LINES 233-234. The text reads: “Availability of antivenom in endemic counties was 41.8%, availability in non-endemic counties was 19.0% (p=0.01).” Better maybe to refer to “highly endemic counties” and “less endemic counties”.

We thank the reviewer for the suggestion. We have now changed the sentence accordingly (results section, p. 14, line 262-263).

6. BOX1. Maybe clarify that the boy received “tetanus toxoid vaccine”. It may be clearer for the reader than just “tetanus toxoid”.

Thank you for the suggestion, we have now changed it to tetanus toxoid vaccine.

7. LINE 255 AND LINE 405. I don’t really like the term “envenoming tests”. It sounds that those are venom-detecting tests, but this is not the case. Would it be possible to use another terminology? ”tests for monitoring of snakebite envenoming” or something like that?

We thank the reviewer for raising this point. We have deleted line 255 (line 287-289) in its entirety as we believe it did not add much to the overall paper, and in line 405 we have omitted the part about envenoming tests (discussion section, p.35, line 450-453).

8. LINE 418. You note that the average recommended dose is 4.5 vials which can go up to 12 vials. Where does that come from? From the product leaflets? Or from the Kenyan guidelines? Please clarify the source and link the text to a reference, if possible.

We have now clarified that it is the average recommended dose based on what manufacturers recommend (discussion section, p.35-36, line 463-467).

9. ONLINE SUPPLEMENT 2. I don’t understand how VINS antivenom can be stocked in 66.67% of all facilities, while it is stocked in 66.67% of facilities in public sector and 100% of facilities in private sector. It should therefore be stocked in more than 66.67% of all facilities… What is the denominator? In addition there is a mismatch between the numbers in Supplemental table 2 and the number in the main text (lines 234-237: “When antivenom was available at facilities, the most commonly stocked brands in the public sector were Snake Venom Antiserum (African IHS) by VINS Bioproducts Ltd (68.4% of facilities), and Inoserp PAN-AFRICAN by INOSAN Biopharma (34.2% of facilities)”.

We thank the reviewer for noting these discrepancies. We have added a footnote to the table explaining that the ‘total’ also includes the availability of the private not-for-profit sector. Further, we have adjusted the erroneous numbers in-text and in the table (results section, p.14, line 263-266), (supporting information table 2).

**Reviewer #2**

The manuscript need minor revision

**Reviewer #3**

-

**Summary and General Comments**

**Reviewer #1**

(No Response)

**Reviewer #2**

1. Major strength of this study is the fact that most of facilities studied were located in rural areas where snakebite is known to be a major public health problem. The study also highlighted important facts regarding snakebite such as; Antivenom is not available in places where it is mostly needed and the fact that in sub-Saharan Africa victims travel long distances to reach health facilities where antivenom is available.

Thank you.

**Reviewer #3**

1. Overall highly relevant and important data on availability of essential stock for treating snakebite envenoming. Commendable use of standardised tools to assess affordability and a strength is that a range of health facilities have been randomly sampled. With above modifications I would suggest accepting this manuscript. A more general comment is that I feel the word count could be trimmed to below 4000. This will increase uptake of readers.

We thank the reviewer for the compliments. Regarding the word count, we’ve critically reviewed our paper again, and found it difficult to cut extensively in the text as it is. The introduction is already quite short (less than 600 words), we reviewed the methods and results and found it difficult to cut those down without loosing important information, leaving the discussion. For the discussion, we believe the current version provides a balanced and rich discussion of important findings, so we would like to suggest to keep all text currently included.
